# Microbiome‐Phytochemical Crosstalk Along the Gut‐Brain Axis in Alzheimer and Parkinson's Disease

**DOI:** 10.1002/mbo3.70374

**Published:** 2026-08-13

**Authors:** Md. Emon, Md. Mizanur Rahaman, Dhrubo Sarder, Md. Shadin, Shamima Akter Neela, Phurpa Wangchuk, Subir Sarker

**Affiliations:** ^1^ Department of Pharmacy Gopalganj Science and Technology University Gopalganj Bangladesh; ^2^ Biomedical Sciences and Molecular Biology, College of Medicine and Dentistry, James Cook University Townsville Queensland Australia; ^3^ Australian Institute of Tropical Health and Medicine, James Cook University Townsville Queensland Australia; ^4^ College of Science and Engineering, James Cook University Cairns Queensland Australia; ^5^ Australian Institute of Tropical Health and Medicine James Cook University Cairns Queensland Australia

**Keywords:** AD, gut microbiota, gut‐brain axis, neuroinflammation, PD, neurodegeneration, phytochemicals, SCFAs

## Abstract

Gut‐brain axis (GBA) has emerged as a bidirectional communication network linking the gut microbiota with central nervous system function and contributing to the pathogenesis of Alzheimer's disease (AD) and Parkinson's disease (PD). This study aimed to critically evaluate the current evidence on how phytochemicals modulate gut microbiota and GBA signaling to influence the progression of AD and PD. A systematic literature search was conducted using major scientific databases, and preclinical studies investigating microbiota‐mediated mechanisms of phytochemicals were synthesized according to predefined inclusion criteria. The available evidence demonstrates that phytochemicals consistently remodel the gut microbiota by increasing beneficial taxa such as *Lactobacillus*, *Bifidobacterium*, *Akkermansia*, and short‐chain fatty acid (SCFA)‐producing bacteria while suppressing pro‐inflammatory Gram‐negative microorganisms. These microbial alterations were associated with reduced lipopolysaccharide (LPS) production, inhibition of TLR4/NF‐κB and NLRP3 inflammasome signaling, enhancement of brain‐derived neurotrophic factor (BDNF) activity, restoration of intestinal barrier integrity, attenuation of oxidative stress, and activation of autophagy‐related pathways, including PI3K/AKT/mTOR and AMPK/mTOR, leading to reduced amyloid‐β, tau, and α‐synuclein pathology. A major contribution of this review is the integration of current evidence demonstrating the bidirectional crosstalk between phytochemicals and the gut microbiota as a central mechanism regulating GBA signaling in AD and PD. Available evidence suggests that phytochemical‐mediated modulation of the gut microbiota represents a promising therapeutic strategy for slowing disease progression in AD and PD. However, further mechanistic investigations and well‐designed clinical trials are required to validate these findings and facilitate their translation into clinical practice.

## Graphical abstract

Phytochemicals remodel the gut microbiota by enriching beneficial bacteria and microbial metabolites while suppressing dysbiosis and inflammation. Through modulation of the gut–brain axis, they restore intestinal barrier integrity, regulate neuroimmune and neuroprotective signaling pathways, reduce amyloid‐β, tau, and α‐synuclein pathology, and ultimately attenuate the progression of Alzheimer's and Parkinson's diseases.

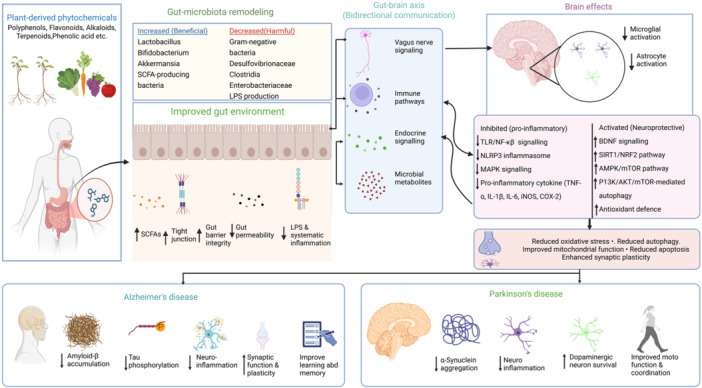

## 1 Introduction

The concept of bidirectional communication between the gastrointestinal tract and the brain now formally recognized as GBA has evolved from early clinical observations to a central paradigm in modern neuroscience and immunology (Zyoud et al. 2019). Over the past decade, the GBA has emerged as a complex, multilayered communication network integrating neural, endocrine, immune, and metabolic signaling pathways (Collins and Bercik 2009). This dynamic crosstalk between the central nervous system (CNS) and the intestinal wall is facilitated and interlocked by an autonomic nervous system (ANS), which communicates via enteric, vagal, and spinal neurons and afferent signals (Chunduri et al. 2022). Although the gut and brain are anatomically separated, they maintain reciprocal communication through neural, endocrine, and inflammatory pathways (Wrona 2006). The vagus nerve acts as the principal channel for GBA, transmitting sensory information from the gut to the brain and modulating motor signals from the brain to the gut, controlling physiological processes like digestion and appetite (Kodidala et al. 2024). Beyond physiological regulation, the GBA plays a pivotal role in maintaining brain homeostasis and shaping emotional, cognitive, and behavioral functions through interactions with the gut microbiota and its bioactive metabolites (Tiwari et al. 2023). Early‐life microbial colonization exerts long‐lasting effects on neural circuit development and synaptic plasticity, whereas microbial dysbiosis has been increasingly associated with CNS dysfunction and neuroinflammatory states (Chen et al. 2013).

Accumulating evidence implicates GBA dysregulation in the pathogenesis of multiple neurodegenerative and neurodevelopmental disorders, including Parkinson's disease (PD) and Alzheimer's disease (AD) (Collins et al. 2012; Bhuiyan et al. 2021). These disorders share a common signaling pathway with GBA, which is influenced by various mechanisms such as pro‐inflammatory cytokines, Aβ accumulation, and gut microbiota (Gupta et al. 2023). Disruption of intestinal barrier integrity may promote systemic immune activation and facilitate peripheral immune cell infiltration into the CNS, thereby amplifying neuroinflammation and accelerating neurodegeneration (Kearns 2024). Moreover, gut microbiota can modulate enteric and central neuronal signaling by regulating receptor expression, neurotransmitter biosynthesis, microglial activation, and cytokine networks involved in disease progression (Lu et al. 2024). Despite strong associative evidence, the precise mechanistic pathways by which GBA perturbations drive neurodegenerative processes remain incompletely understood.

In parallel, bioactive compounds derived from plants have gained considerable attention for their pleiotropic pharmacological properties, including antioxidant, anti‐inflammatory, immunomodulatory, and neuroprotective effects (Kurhekar 2021; Geronikaki and Gavalas 2006). Emerging evidence suggests that these compounds exert systemic benefits not only through direct CNS activity but also through modulation of the GBA (Kim et al. 2024). Jaberi et al. (2024), comprehensively evaluated the capacity of phytochemicals to modulate GBA signaling, highlighting mechanistic links between dietary bioactive and neurophysiological regulation. Abbaszadeh et al. (2025), further expanded this perspective by identifying phytochemicals derived from edible flowers with potential neuroprotective properties mediated through gut–brain communication. Consistently, Wang et al. (2022a), underscored the therapeutic promise of dietary phytochemicals as anti‐Alzheimer's agents, primarily through microbiota‐dependent pathways.

Beyond phytochemical‐focused analyses, Liaqat et al. (2022), delineated the contribution of gut microbiota dysbiosis to PD pathogenesis, while Li Yueqin et al. emphasized the regulatory influence of polyphenols on GBA‐associated molecular pathways in neurodegenerative diseases (NDDs). More broadly, accumulating evidence from foundational and contemporary syntheses has reinforced the central role of the microbiota–gut–brain axis in neurodegeneration Loh et al. (2024), Westfall et al. (2017), and Ghaisas et al. (2016) have collectively reinforced the central role of the microbiota–gut–brain axis in neurodegeneration.

Given the breadth of neurodegenerative disorders, the present review deliberately focuses on AD and PD, the two most extensively investigated disorders in which gut microbiota‐phytochemical interactions have been mechanistically characterized. By narrowing the scope, this review aims to critically synthesize evidence describing how phytochemicals modulate gut microbiota and gut–brain axis signaling to influence disease pathogenesis and progression. Specifically, we discus, (i) microbiota‐mediated mechanisms of phytochemicals, (ii) gut‐brain axis signaling pathways involved in AD and PD, and (iii) current challenges and future directions for translating these findings into clinical practice.

## 2 Materials and Methods

### 2.1 Search Strategy

A structured literature search was conducted to identify original studies investigating the interaction between phytochemicals, gut microbiota, and the gut–brain axis in neurodegenerative disease specifically AD and PD. Electronic databases including PubMed, Scopus, Web of Science, and Google Scholar, were systematically searched till January 2026. The literature search included studies published between January 2004 and December 2025 to ensure comprehensive coverage of both foundational and recent advances in gut microbiota, phytochemicals, and gut–brain axis research. The search strategy combined Medical Subject Headings (MeSH) and free text keywords, using Boolean operators. Representative search terms included:

*(“gut–brain axis” OR “gut microbiota” OR “microbiome” OR “intestinal microbiota”) AND (“phytochemicals” OR “plant‐derived compounds” OR “polyphenols” OR “flavonoids”) AND (“neurodegenerative disease” OR “Alzheimer's disease” OR “Parkinson's disease”)*. Reference lists of all eligible articles were manually screened to identify additional relevant studies not retrieved during the electronic database search.

### 2.2 Eligibility Criteria

Studies were selected according to predefined inclusion and exclusion criteria.

#### 2.2.1 Inclusion Criteria

Studies were included if they:

(i) investigated phytochemicals or plant‐derived bioactive compounds, (ii) evaluated gut microbiota modulation, microbial metabolites or GBA‐related mechanism,

(iii) examined neurodegenerative disease specifically AD and PD using in vitro, animal, or clinical studies.

(iv) reported mechanistic evidence linking phytochemicals with gut microbiota‐mediated neuroprotection; and

(v) were published in peer‐reviewed journals in English between January 2010 and December 2025.

#### 2.2.2 Exclusion Criteria

Studies were excluded if they:

(i) were review articles, editorials, and conference abstracts, book chapters, letters, or commentaries;

(ii) studies lacking microbiota or GBA‐related outcomes;

(iii) focused exclusively on phytochemical pharmacology without investigating microbiota‐related mechanisms;

(iv) reports without mechanistic relevance to neuroinflammation, microbial metabolites, or neuroprotection.

### 2.3 Study Selection and Data Extraction

**Figure 1:**
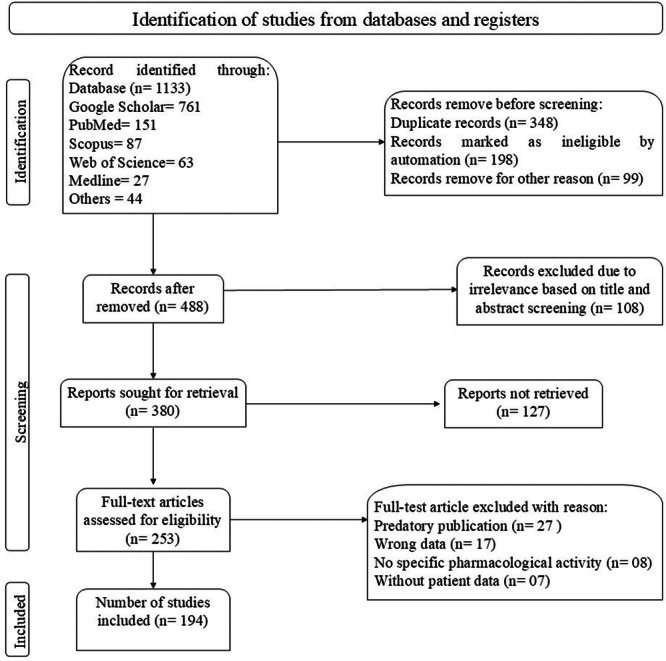
PRISMA flow‐diagram based on data extraction.

Following the removal of duplicate records, the titles and abstracts of all retrieved articles were screened for relevance according to the predefined eligibility criteria. Potentially eligible studies subsequently underwent full‐text assessment to determine their suitability for inclusion in the review. The study selection process is summarized in Figure 1. Data from the eligible studies were extracted using a standardized data extraction framework to ensure consistency and accuracy throughout the review process. The extracted information included the first author and year of publication, phytochemical or plant‐derived bioactive compound investigated, experimental model (in vitro, animal, or clinical), disease model (AD or PD), treatment dose and duration (where available), alterations in gut microbiota composition, microbial metabolites, gut–brain axis‐related signaling pathways, inflammatory and oxidative stress biomarkers, and the principal neuroprotective outcomes. The extracted data were subsequently synthesized to compare mechanistic findings across studies and to identify common molecular pathways through which phytochemicals modulate the gut microbiota and influence gut–brain axis communication in AD and PD.

### 2.4 Quality Assessment

**Figure 2:**
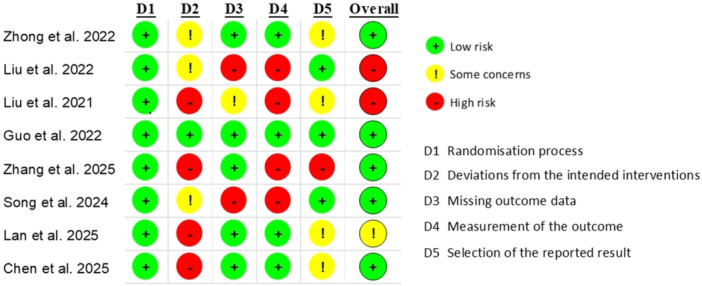
Risk of bias assessment. According to the Cochrane risk‐of‐bias tool for randomized trials (RoB 2) Interventions, +: low risk of bias; −: high risk of bias; !: some concern risk of bias.

The methodological quality of the included studies was critically assessed based on study design, experimental model (in vitro, animal, or clinical), sample size, mechanistic evidence, reproducibility of findings, and translational relevance. Particular consideration was given to potential sources of bias, including limitations associated with preclinical animal models, small sample sizes, and inconsistencies among studies. Greater emphasis was placed on well‐designed mechanistic and clinical investigations during evidence interpretation. To further evaluate the methodological quality of the included evidence, a risk‐of‐bias assessment was performed using the Cochrane Risk of Bias (RoB 2) tool (version 2, August 22, 2019), and the results are presented in Figure 2.

### 2.5 Data Synthesis

Because of substantial heterogeneity in phytochemical classes, experimental models, microbiota analyses, and outcome measures, a quantitative meta‐analysis was not performed. Therefore, findings were synthesized qualitatively.

The synthesis emphasized common mechanistic pathways through which phytochemicals modulate gut microbiota and influence gut–brain axis communication. Particular focus was placed on microbial compositional changes, short‐chain fatty acid production, intestinal barrier integrity, neuroimmune interactions, inflammatory signaling pathways (TLR4/NF‐κB, NLRP3 inflammasome, MAPK, and JAK/STAT), neurotrophic regulation (BDNF), oxidative stress (Nrf2/HO‐1), neurotransmitter metabolism, mitochondrial function, autophagy, and neurotrophic signaling, including BDNF.

Conflicting findings, methodological limitations, and differences between in vitro, animal, and clinical studies were critically compared to providing a balanced interpretation of the current evidence and identifying important knowledge gaps.

## 3 Results and Discussion

### 3.1 Pathophysiology of PD and AD

PD and AD are the two most prevalent NDDs, characterized by the progressive loss of neuronal structure and function, ultimately leading to neuronal death (Emerit et al. 2004). A defining hallmark of these disorders is the abnormal aggregation of misfolded proteins, including amyloid‐β (Aβ) and tau in AD, α‐synuclein in PD (Sengupta and Kayed 2022). These misfolded and aggregated proteins exert cytotoxic effects, notably impairing synaptic plasticity, which contributes to neuronal dysfunction and degeneration (Tian et al. 2020).

Protein aggregates also play a critical role in activating immune responses within the central nervous system. In particular, they stimulate microglia, the resident immune cells of the brain, leading to the enhanced production of pro‐inflammatory mediators such as TNF‐α, IL‐1β, and IL‐6, thereby promoting neuroinflammation (Tian et al. 2020). Under physiological conditions, microglial activation serves protective functions, including the clearance of cellular debris and the maintenance of tissue homeostasis (Woodburn et al. 2021). However, in ageing individuals, microglial dysfunction may result in an exaggerated and sustained pro‐inflammatory response to the same stimuli, fostering chronic neuroinflammation and accelerating neuronal degeneration in both AD and PD (Angelova and Brown 2019).

Oxidative stress represents another key pathogenic mechanism underlying both AD and PD and is closely associated with protein misfolding (Reynolds et al. 2007). It arises from an imbalance between the production of reactive oxygen species (ROS) and the capacity of endogenous antioxidant defense systems (Bhattacharya 2014). Elevated ROS levels induce oxidative modifications in proteins, including tau, α‐synuclein, and Aβ, thereby increasing their propensity for misfolding and aggregation (Pedersen et al. 2016).

Notably, depressive disorders frequently co‐occur with both AD and PD, likely reflecting shared underlying pathophysiological mechanisms such as neuroinflammation and oxidative stress (Alachkar et al. 2022). Epidemiological evidence further indicates that anxiety and depression affect more than 40% of patients with AD and PD, underscoring the clinical relevance of these interconnected processes (Lian et al. 2024; Botto et al. 2022).

### 3.2 Gut Microbiota in AD and PD

Growing evidence indicates that gut microbiota plays a critical role in the pathogenesis of AD and PD through GBA has been increasingly recognized (Iyer et al. 2024). Gut microorganisms influence neurotransmitter homeostasis through several mechanisms. Gut microbiota synthesizes and modulates a wide range of chemical messengers that influence CNS function via neural, endocrine, and circulatory pathways, including signaling through the vagus nerve (Kuwahara et al. 2020). Notably, gut microbes can produce, regulate, or metabolize key neurotransmitters such as 5‐hydroxytryptamine (5‐HT), dopamine, glutamate, norepinephrine, and γ‐aminobutyric acid (GABA) (Mazzoli and Pessione 2016). Certain bacteria synthesize neurotransmitters directly, whereas others regulate the availability of their metabolic precursors or stimulate host intestinal cells to produce neuroactive molecules. For instance, spore‐forming *Clostridia* activates enterochromaffin cells to produce 5‐HT from tryptophan (Mandić et al. 2019), whereas *Lactobacillus* and *Enterococcus* facilitate the conversion of tyrosine to l‐DOPA (Song et al. 2025), thereby modulating dopaminergic neurotransmission. Similarly, *Bifidobacterium* converts glutamine to glutamate, modulating glutamatergic activity (Royo et al. 2023). Norepinephrine biosynthesis is indirectly regulated through microbial control of tyrosine availability (Sudo 2019) and HPA axis modulation (Rusch et al. 2023), while *Lactobacillus rhamnosus* synthesizes GABA via glutamate decarboxylase, transmitting effects centrally through the vagus nerve (Tette et al. 2022). Dysregulation of these neurotransmitters have been shown to be closely associated with the onset and progression of NDDs (Teleanu et al. 2022).

Microbiota‐derived signaling plays a fundamental role in several neurological processes, including neurotransmission, dendritic development, and synaptic plasticity (Maqsood and Stone 2016). Emerging evidence further suggests that the intestinal microbiota contributes to microglial maturation and activity, thereby facilitating the clearance of neurotoxic protein aggregates through phagocytic mechanisms (Fu et al. 2014). In addition, the gut microbiota influences the expression of BDNF, a neurotrophin essential for neuronal survival, differentiation, and synaptic function, which is frequently dysregulated in NDDs (Sharma et al. 2023).

An important functional output of the gut microbiota is the production of short‐chain fatty acids (SCFAs), which modulate host immune responses by influencing interleukin release and are implicated in the regulation of neuroinflammation and neurogenesis (Guo et al. 2025). Conversely, the gut microbiota may also participate in pro‐degenerative signaling pathways, including activation of the inflammasome and nuclear factor kappa B (NF‐κB) cascades (Zhang et al. 2022a). Gut‐microbiota derived molecules, such as lipopolysaccharide (LPS) activates NF‐κB‐mediated neuroinflammation which upregulates neuroinflammatory microRNAs, contributing to neuronal atrophy. Moreover, Gut dysbiosis promotes the overproduction of microbial amyloids by gram‐negative bacteria, cross‐seeding misfolding of amyloid‐β and α‐synuclein while activating microglia (Welcome 2019). These processes can contribute to sustained neuroinflammation, directly accelerating AD and PD pathology (Mou et al. 2022). Overall, these findings underscore the gut microbiota as an important contributor to the pathogenesis of AD and PD and a promising therapeutic target for modulating GBA dysfunction.

### 3.3 Roles of Phytochemicals in AD and PD

Over the past decades, increasing attention has been given to the therapeutic potential of plant‐derived bioactive compounds in AD and PD (Singh et al. 2024a). Emerging evidence suggests that phytochemicals including polyphenols, flavonoids, alkaloids, carotenoids, and plant steroids exhibit significant neuroprotective effects, not only through antioxidant and anti‐inflammatory, activities but also by reshaping gut microbiota composition and microbial metabolite production (Shimizu 2017). Through interaction with the GBA, this modulation influence neuroinflammation, protein aggregation, intestinal barrier integrity, and neuronal signaling, making microbiota–phytochemical interactions a promising therapeutic strategy for AD and PD (Hong et al. 2023).

The clinical relevance of natural compounds is underscored by the therapeutic application of several plant‐derived molecules. For instance, physostigmine, originally isolated from the Calabar bean, has been utilized in the management of AD, while morphine, derived from the opium poppy, remains a well‐established agent in neurological and inflammatory conditions (Jee et al. 2025). More recently, numerous phytochemicals have demonstrated neuroprotective potential in experimental models of AD and PD by modulating gut microbiota composition, suppressing neuroinflammation, reducing oxidative stress, and regulating protein aggregation (Jaberi et al. 2024; Kustrimovic et al. 2024; Gupta and Sharma 2026). These examples highlight the translational potential of phytochemicals as therapeutic agents. Thus, phytochemicals represent a promising strategy for the prevention and management of AD and PD through modulation of gut microbial dynamics and gut–brain axis signaling (Babazadeh et al. 2020). The potential mechanisms through which phytochemicals influence NDDs via the GBA are summarized below.

#### 3.3.1 Parkinson's Disease (PD)

PD has traditionally been considered a central nervous system disorder, accumulating evidence now implies that the gastrointestinal dysfunction and gut microbiota dysbiosis are critical contributor to PD pathogenesis, reinforcing the relevance of GBA interactions (Ferrer 2009; Warnecke et al. 2022). A central pathological feature of PD is the aggregation of α‐synuclein (aSyn), which has been closely associated with GBA dysfunction; emerging studies indicate that phytochemicals may mitigate aSyn aggregation, partly through modulation of gut microbial populations, including suppression of pro‐inflammatory or amyloid‐producing bacteria such as *Escherichia coli*, *Shigella*, and *Salmonella* (Sampson et al. 2020; Miller et al. 2021).

**Figure 3:**
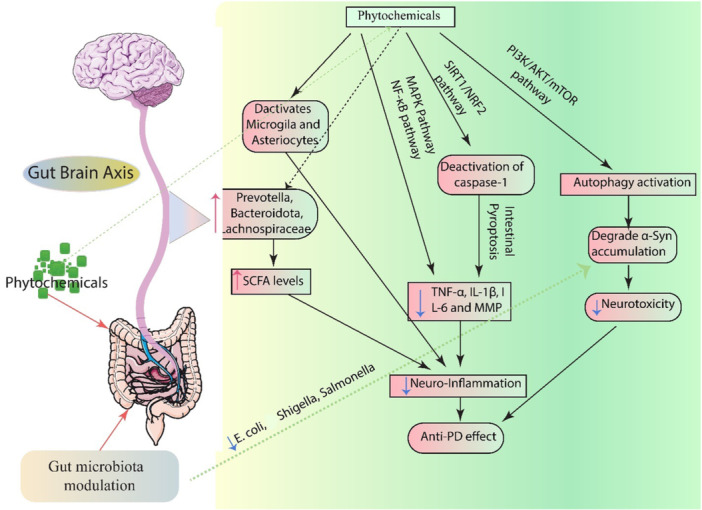
Schematic representation of how phytochemicals modulate gut microbiota and host signaling pathways to mitigate PD. Phytochemicals increase beneficial microbes and SCFAs while reducing pathogenic bacteria, leading to improved intestinal barrier function and immune regulation. They modulate key pathways, including NF‐κB, MAPK, SIRT1/NRF2, and PI3K/AKT/mTOR, resulting in reduced microglial and astrocyte activation, decreased pro‐inflammatory cytokines, and inhibition of caspase‐1 mediated pyroptosis. Enhanced autophagy promotes α‐synuclein clearance and reduces neurotoxicity and contributes to anti‐PD effects.

Phytochemicals induced remodeling of the gut microbiota also influences intracellular signaling pathways relevant to protein homeostasis. Phytochemical‐mediated modulation of the gut microbiome further increased the SCFAs producing taxa, such as *Prevotella*, members of *Bacteroidota*, and *Lachnospiraceae* (Tremlett et al. 2017; Lin et al. 2018). Elevated SCFAs levels contribute to immune regulation and maintenance of intestinal barrier integrity, processes that are intimately linked to neuroinflammatory responses observed in PD (Aho et al. 2021). These microbiota‐mediated effects are accompanied by modulation of the PI3K/AKT/mTOR signaling cascade, resulting in promotes autophagic and clearance of aggregated aSyn (Yu et al. 2024). Through modulation of gut microbial composition, phytochemicals reduce intestinal inflammation and limit the translocation of pro‐inflammatory microbial products. Consequently, activation of microglia and astrocytes is attenuated, leading to reduced production of TNF‐α, IL‐1β, IL‐6, and matrix metalloproteinases (MMPs), thereby suppressing neuroinflammatory cascades associated with PD (Davinelli et al. 2016; Kempuraj et al. 2016). Recent experimental studies using TNBS model of colitis demonstrated that phytochemical treatment remodels gut microbial composition by enriching beneficial bacterial taxa while reducing pro‐inflammatory microorganisms, supporting the concept that microbiota modulation contributes to the anti‐inflammatory effects of phytochemicals (Rahaman et al. 2025a). phytochemicals also modulate key signaling pathways implicated in inflammation and oxidative stress, including NF‐κB, SIRT1/NRF2, and MAPK pathways (Flemming et al. 2015; Liu and Li 2019). Specifically, activation of the SIRT1/NRF2 axis has been associated with reduced intestinal pyroptosis via deactivation of caspase‐1, further highlighting the cytoprotective potential of phytochemicals (Zhong et al. 2022). These interconnected roles are summarized in Figure 3 and Table 1.

**Table 1:** Data of phytochemicals effective against Parkinson disease.

Compound	Dose	Experimental model	Animal model	Gut microbiota modulation	Mechanism of action on gut–brain axis	Reference
Curcumin	60 mg/kg	MPTP‐induced PD model	Male C57BL/6 J mice	↑ Prevotella; ↓ Enterobacteriaceae	Activates SIRT1/NRF2 signaling, attenuates intestinal inflammation and oxidative stress	(Zhong et al. (2022))
Piperine	20 mg/kg	6‐OHDA‐induced PD model	Male Sprague‐Dawley rats	↓ Bacteroides, Prevotella, Salmonella, Escherichia	Activates autophagy via PI3K/AKT/mTOR inhibition; promotes α‐synuclein degradation	(Yu et al. (2024))
Fisetin	100 mg/kg	MPTP model	Male C57BL/6 mice	↑ Akkermansia muciniphila; ↓ Escherichia‐Shigella	dysbiosis‐	(Chen et al. (2020))
Fucosylated chondroitin sulfate	50 mg/kg	MPTP model	Mice	↓ Muribaculaceae; ↑ Staphylococcus	Suppresses NF‐κB signaling; reduces gut‐derived inflammation	(Liu et al. (2022a))

#### 3.3.2 Alzheimer's Disease (AD)

**Figure 4:**
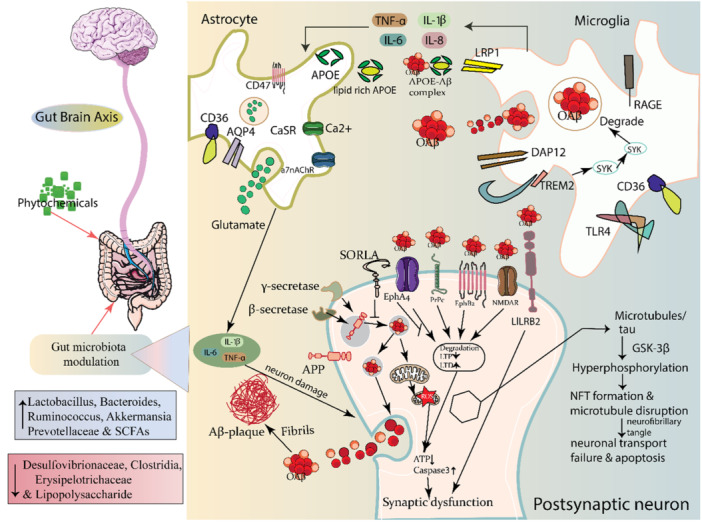
Overview of the interactions between phytochemicals, gut microbiota, glial cells, and neuronal signaling pathways in AD. Phytochemicals promote beneficial bacteria (*Lactobacillus*, *Bacteroides*, *Ruminococcus*, *Akkermansia*, and *Prevotellaceae*) and increase SCFAs production while reducing pro‐inflammatory taxa (e.g., *Desulfovibrionaceae*, *Clostridia*, and *Erysipelotrichaceae*) and LPS levels. SCFAs suppress NLRP3 inflammasome activation and inflammatory cytokine release. Within the brain, phytochemicals modulate APP processing, autophagic degradation pathways, oxidative stress, and mitochondrial dysfunction, thereby reducing amyloid‐β (Aβ) accumulation. The diagram also highlights astrocyte–microglia communication involving APOE‐lipid complexes, LRP1‐mediated transport, and receptors such as TREM2, CD36, TLR4, and RAGE that regulate Aβ uptake and degradation. Downstream neuronal consequences include reduced tau hyperphosphorylation, inhibition of GSK‐3β signaling, prevention of neurofibrillary tangle formation, improved synaptic function, and decreased apoptosis. Together, gut microbiota remodeling and neuroprotective signaling pathways contribute to attenuation of AD pathology.

Gut dysbiosis is increasingly recognized as an important contributor to AD pathogenesis through disruption of gut–brain axis signaling (Figure 4). A shift in gut microbiota composition is characterized by a reduction in beneficial taxa such as Lactobacillus, Bacteroides, and Akkermansia, alongside their protective SCFAs, and an expansion of pro‐inflammatory species including members of Desulfovibrionaceae and Clostridia coupled with increased LPS, which promotes systemic inflammation and neuroimmune signaling. These microbiota‐derived signals activate microglia and astrocytes, triggering the release of pro‐inflammatory cytokines, including TNF‐α, IL‐1β, IL‐6, and IL‐8, thereby promoting amyloid precursor protein (APP) processing, amyloid‐β (Aβ) accumulation, tau hyperphosphorylation through glycogen synthase kinase‐3β (GSK‐3β), mitochondrial dysfunction, synaptic impairment, and ultimately neuronal apoptosis (Gouras et al. 2005). Amyloid‐β (Aβ) accumulation is widely recognized as a central pathological feature of AD (Gouras et al. 2005). phytochemicals have been shown to promote the autophagic clearance of Aβ via modulation of the AMPK/mTOR signaling pathway (Cheng et al. 2024). In parallel, phytochemicals downregulate APP, the primary source of Aβ plaque formation (Liu et al. 2021). Emerging evidence further suggests that phytochemical‐mediated remodeling of the gut microbiota contributes to these effects by increasing the abundance of beneficial bacterial taxa and their metabolites. Notably, APP expression may also be indirectly reduced through elevations in gut‐derived SCFAs (Zhang et al. 2023). SCFAs, including acetate, propionate, isobutyrate, butyrate, and valerate have been reported to interfere with the aggregation of Aβ_1_–_40_ and Aβ_1_–_42_ peptides, thereby mitigating amyloidogenic processes (Ho et al. 2018).

Phytochemicals exert substantial effects on gut microbial composition, favoring the expansion of beneficial taxa such as *Lactobacillus*, *Bacteroides*, *Ruminococcus*, *Akkermansia*, and members of *Prevotellaceae*, which contribute to enhanced SCFAs production (Liu et al. 2018). SCFAs are negatively associated with NLRP3 inflammasome‐mediated neuroinflammation (Shen et al. 2020). Activation of the NLRP3 inflammasome leads to caspase‐1 activation, which triggers the release of IL‐1β and IL‐6, resulting in neuroinflammation (Flores et al. 2022). LPS, a pro‐inflammatory neurotoxin produced by many Gram‐negative bacteria in the gastrointestinal tract, also stimulates the release of TNF‐α, IL‐1β, and IL‐2 via the NF‐κB pathway (Batista et al. 2019). Phytochemicals mediated remodeling of the gut microbiota may reduce the abundance of pro‐inflammatory Gram‐negative taxa, including members of *Desulfovibrionaceae*, *Clostridia*, and *Erysipelotrichaceae*, thereby attenuating inflammatory burden (Liu et al. 2022b).

Moreover, phytochemicals reduce microglial hyperactivation through the cytokine signaling inhibitor 1 pathway, thereby further suppressing neuroinflammation (Jo et al. 2019). Beneficial bacteria promoted by phytochemicals also help repair damage to the gut barrier by upregulating the expression of Muc‐2 and tight junction proteins (Zhan et al. 2024). This strengthens gut integrity, improves BBB function, and reduces the accumulation of Aβ and tau proteins in the brain (Liu et al. 2020). In addition, phytochemicals have demonstrated cholinergic regulatory effects, including increased serum acetylcholine (ACh) levels and choline acetyltransferase (ChAT) activity, alongside reduced acetylcholinesterase (AChE) activity. These changes collectively support improved cholinergic neurotransmission and cognitive performance (Ren et al. 2020). These findings demonstrate that phytochemicals mitigate AD progression by remodeling gut microbiota, enhancing SCFAs production, suppressing neuroinflammation, restoring gut and blood–brain barrier integrity, and regulating amyloidogenic, tau‐related, and cholinergic signaling pathways. These integrated microbiota–gut–brain axis interactions highlight the therapeutic potential of phytochemicals in AD, as illustrated in Figure 4, while the diversity of phytochemicals investigated in AD is summarized in Table 2.

**Table 2:** Data of crude extract and phytochemicals effective against Alzheimer's disease.

Compound/extract	Dose	Experimental model	Animal model	Gut microbiota modulation	Mechanism of action on gut–brain axis	Reference
Sesamol	100 mg/kg	APP/PS1 model	Male APPswe/PS1dE9 mice	↑ Bifidobacterium; ↓ Helicobacter hepaticus, Clostridium	Inhibits microglial overactivation; decreases TNF‐α and IL‐1β	Liu et al. (2021)
Turmeric powder	80 mg/kg	DSS‐induced model	Mice	↑ Lactobacillus, Bifidobacterium	Activates AhR signaling; enhances tight junction proteins and IL‐22 expression	Yang et al. (2022)
Berberine	100–200 mg/kg	Aβ‐induced model	5xFAD mice	↓ Bacillus thuringiensis, Bacteroides anthropophilus	Clears Aβ plaques; reduces neuroinflammation	Sun et al. (2024)
Salidroside	50 mg/kg	LPS‐induced model	Male SAMP8 mice	↓ Clostridiales, Streptococcaceae	Reduces Aβ1‐42 deposition; suppresses IL‐1β, IL‐6, TNF‐α	Xie et al. (2020)
Isoorientin	50 mg/kg	AD biomarker model	APP/PS1 mice	↓ Gram‐negative bacteria (Alteromonas, Campylobacterales)	Inhibits LPS‐mediated NF‐κβ activation	Zhang et al. (2022a)
Silibinin	100 mg/kg	APP/PS1 model	Male APP/PS1 mice	↑ Bacteroidetes, Firmicutes	Reduces amyloid burden and memory deficits	Shen et al. (2019)
Triphala	500 mg/kg	Antibiotic‐treated AD model	5XFAD mice	↑ Bacteroidetes, Verrucomicrobiota; ↓ Proteobacteria	Increases butyrate; restores gut dysbiosis	Upadhyay and Gupta (2023)
β‐Glucan	100 mg/kg	HFD model	Female SD rats	↑ Akkermansia, Ruminococcaceae	SCFA‐mediated inhibition of NLRP3 inflammasome	Mo et al. (2024)
Ginkgo biloba	100 mg/kg/day	APP/PS1 model	Male APP/PS1 mice	↑ Bacteroidetes; ↓ Firmicutes	Regulates microbial metabolism	Yu et al. (2023)
Camellia oil	1 mL/kg/day	Aβ25‐35 model	Male Kunming mice	↓ Bacteroidetes; ↑ Firmicutes	Reduces ROS; increases GSH‐Px and SOD	Guo et al. (2022)
Palmatine	0.6 mg/mL	Aβ1‐40 model	Male SD rats	↑ Firmicutes, Bacteroidetes	Improves brain and gut tissue morphology	Han et al. (2024)
Hesperetin	50 mg/kg	HFD + STZ model	Male SD rats	↑ SCFA‐producing bacteria	Activates BDNF signaling pathway	Zhang et al. (2025)
Catechin	100 mg/kg	HFD‐induced model	Male C57BL/6 J mice	↑ Alloprevotella, Muribaculaceae	Modulates PPAR‐γ/NF‐κβ signaling; regulates dopamine and TNF‐α levels	Zhou et al. (2023)
Theaflavin	100 mg/kg	CUMS model	Mice	↓ Bacteroidetes, Lachnospiraceae	Enhances antioxidant capacity; increases neurotrophic factors	Li et al. (2023)
Baicalein	50 mg/kg	BCCAO model	Male SD rats	↑ Firmicutes; ↓ Bacteroidetes	Inhibits TLR4/MyD88/NF‐κB pathway	Song et al. (2024)
Chlorogenic acid	150 mg/kg	HFFD model	C57BL/6 J mice	↑ Lactobacillus, Bifidobacterium; ↓ Ruminococcaceae	Enhances synaptic transmission; reduces neuroinflammation	Zhang et al. (2022b)
Quinoa	500 mg/kg	Hypoxia model	Mice	Modulates Bacteroidetes, Actinobacteria	Restores gut barrier; increases SCFAs	Lan et al. (2025)
Vitexin	10 mg/kg	HFD model	Male C57BL/6 N mice	↑ Akkermansia, Lachnospiraceae	Exerts antioxidant and anti‐inflammatory effects	Li et al. (2021a)
Cyanidin‐3‐glucoside	50 mg/kg	Tribromoethanol model	Male C57BL/6 J mice	↑ Faecalibaculum, Bifidobacterium	Activates ERK/CREB/BDNF pathway	Chen et al. (2025)
Blackcurrants	308 mg/day	MTF model	Adult females (human study)	↑ Bifidobacterium longum, B. bifidum	Enhance memory via microbiota modulation	Gillies et al. (2024)

### 3.4 Impact on the Gut‐Brain Axis

#### 3.4.1 Microbiota

The gut microbiota has been described as a hidden organ closely connected to multiple illnesses such as obesity, diabetes, inflammatory bowel disease, cardiovascular disorders, and NDDs, particularly AD and PD (Wang et al. 2022b; Schroeder and Bäckhed 2016; Rahaman et al. 2025b, 2025a). Phytochemicals modulate gut microbiota by altering both microbial composition and metabolic activity (Yin et al. 2019). These bioactive compounds promote the growth of beneficial taxa such as *Lactobacillus*, *Bifidobacterium*, *Prevotellaceae*, and *Akkermansia muciniphila*, while concurrently suppressing potentially harmful populations including *Desulfovibrionaceae*, *Faecalibaculum*, *Clostridia*, and *Erysipelotrichaceae* (Rahaman et al. 2025a; Liu et al. 2022a, 2022b, 2019). Beneficial gut microbiota enhances the production of SCFAs, including acetate, propionate, isobutyrate, butyrate, and valerate, while simultaneously reducing LPS levels and modulating inflammatory cytokines (Fernández et al. 2016), thereby improving intestinal barrier integrity, suppressing neuroinflammation, and maintaining gut–brain axis homeostasis in AD and PD.

In contrast, dysbiotic bacteria promote toxic metabolite accumulation, elevate LPS, and trigger excessive pro‐inflammatory signaling, eading to chronic activation of microglia and astrocytes through gut–brain axis communication. These processes contribute to amyloid‐β and tau pathology in AD, as well as α‐synuclein aggregation and dopaminergic neuronal degeneration in PD (Di Vincenzo et al. 2024). By shaping microbial composition, phytochemicals influence gut‐derived signaling pathways that regulate neuroinflammation, protein aggregation, and neuronal survival, thereby slowing the progression of AD and PD (Zhong et al. 2023).

#### 3.4.2 Short‐Chain Fatty Acids (SCFAs)

Phytochemicals enhance the abundance of SCFA‐producing bacteria in the gut (Singh et al. 2024b). SCFAs are small organic monocarboxylic acids with six or fewer carbon atoms, including acetate, propionate, butyrate, valerate, and their isoforms (Rekha et al. 2024). They represent the predominant metabolites of the gut microbiota, produced through the anaerobic fermentation of indigestible dietary polysaccharides in the large intestine (Ji et al. 2024).

Emerging evidence indicates that SCFAs exert potent anti‐inflammatory and neuroprotective effects through multiple interconnected molecular mechanisms that are highly relevant to AD and PD. At the transcriptional level, SCFAs suppress the NF‐κB signaling pathway by blocking IκB kinase (IKK) phosphorylation, which preserves IκBα and prevents NF‐κB nuclear translocation, thereby suppressing transcription of TNF‐α, IL‐1β, IL‐6, and IL‐8 (Zheng et al. 2025; Abdelazeem et al. 2021). In addition, butyrate attenuates NLRP3 inflammasome activation, reducing caspase‐1‐mediated maturation and release of IL‐1β and IL‐18 (Park et al. 2023). SCFAs enhance intestinal barrier integrity by regulating tight junction proteins, thereby limiting LPS translocation and TLR4‐mediated inflammation (Liu et al. 2023a). Through gut–brain axis communication, these anti‐inflammatory effects reduce microglial activation and contribute to the attenuation of neuroinflammation associated with AD and PD.

In addition, SCFAs interfere with the formation of toxic protein aggregates such as amyloid‐β and α‐synuclein and enhance GABA levels, thereby reducing neuronal hyperexcitability and supporting neuronal survival (Oleskin and Shenderov 2016).

#### 3.4.3 Lipopolysaccharide (LPS)

LPS is an immunostimulatory endotoxin component of the outer membrane of Gram‐negative bacteria (Wang and Quinn 2010) (Rosendo‐Silva et al. 2023). In a healthy gut, gram‐negative bacteria exist in a balanced microbial community and tight junction proteins (claudin, occludin, ZO‐1) maintain intestinal barrier integrity, thereby restricting LPS to the gut lumen (Di Vincenzo et al. 2024). During dysbiosis, overgrowth of gram‐negative bacteria together with a decline in SCFA‐producing bacteria results in increased intestinal LPS production (Roy et al. 2025). Following systemic translocation, LPS binds to LPS‐binding protein (LBP), which facilitates its transfer to the TLR4 receptor complex on monocytes and macrophages (Luo et al. 2025). Activation of TLR4 triggers the MyD88‐dependent signaling pathway, leading to phosphorylation of IKKβ. Activated IKKβ subsequently phosphorylates IκBα, resulting in NF‐κB activation and nuclear translocation, which drives the transcriptional upregulation of pro‐inflammatory cytokines, including TNF‐α, IL‐1β, IL‐6, and IL‐8 (Yin et al. 2025).

Phytochemical‐mediated remodeling of the gut microbiota increases the abundance of SCFA‐producing bacteria while reducing LPS‐producing Gram‐negative taxa, thereby restoring gut microbial homeostasis. SCFA depletion and elevated LPS levels represent mechanistically linked consequences of a common dysbiotic state (Weiss and Hennet 2017). SCFAs, particularly butyrate, strengthen intestinal barrier integrity by upregulating tight junction proteins, thereby preventing LPS translocation into the systemic circulation and inhibiting IKK phosphorylation, which suppresses downstream TLR4/NF‐κB signaling (Gao et al. 2025).

Consequently, reduced LPS‐mediated activation of microglial attenuates chronic inflammation, oxidative stress, and synaptic dysfunction, thereby limiting amyloid‐β and tau pathology in AD and α‐synuclein aggregation in PD (Kumar et al. 2020).

#### 3.4.4 Neurotransmitters

Gut microbiota directly synthesizes or modulates neurotransmitters, including GABA, serotonin (5‐HT), dopamine, acetylcholine, and glutamate (Kumar et al. 2020). These neuroactive compounds are produced either directly by gut microorganisms or indirectly through microbial regulation of neurotransmitter precursors and host enteroendocrine cells, thereby influencing gut–brain axis signaling. Probiotics including *Lactobacillus* increase GABA levels in the small intestine, while *Bifidobacteria* have been shown to elevate fecal GABA concentrations in healthy individuals (Kaur et al. 2023). Interestingly, these increases were negatively correlated with glutamate concentrations, highlighting the contribution of GABAergic activity in AD and PD (Czapski and Strosznajder 2021). Phytochemicals enhance the abundance of *Lactobacillus* and *Bifidobacteria* abundance, thereby increasing GABA availability via the GBA (Peterson 2020). These GABA‐producing gut bacteria synthesize GABA directly from glutamate via the enzyme glutamate decarboxylase.

In contrast, glutamate, the main excitatory neurotransmitter in the central nervous system, is essential for neuronal function but becomes neurotoxic when dysregulated in AD and PD (Iovino et al. 2020). Excessive glutamate and reduced glutamine impair neurotransmitter cycling, causing excitotoxicity, oxidative stress, and neuronal injury, as observed in Alzheimer's and Parkinson's diseases (Quincozes‐Santos et al. 2014). Impaired astrocytic glutamine synthetase further promotes glutamate accumulation, leading to synaptic dysfunction and neuroinflammation (Zou et al. 2010). Phytochemicals mitigate these effects by lowering glutamate levels.

Similarly, serotonin dysregulation has been implicated in PD and AD (Teleanu et al. 2022; Shabbir et al. 2013). Notably, approximately 90‐95% of serotonin in the body is synthesized in the gut by enterochromaffin cells, underscoring its critical role in gut physiology and brain communication (Jones et al. 2020). Phytochemicals remodel the gut microbiota, particularly by enriching *Lactobacillus* species, which promotes serotonin synthesis from tryptophan in enterochromaffin cells and modulates gut–brain axis and signaling, thereby exerting neuromodulatory effects in AD and PD (Afshari et al. 2020; Rebas et al. 2020). Moreover, phytochemicals enhance acetylcholine levels and improve cholinergic transmission by promoting the fermentation‐driven production of butyrate, which upregulates ChAT expression and expands the cholinergic neuron population within the enteric nervous system.

#### 3.4.5 Brain‐Derived Neurotrophic Factor (BDNF)

BDNF, a member of the neurotrophin family of growth factors, which also includes nerve growth factor (NGF), neurotrophin‐3 (NT‐3), and neurotrophin‐4 (NT‐4), plays a critical role in neuronal survival, differentiation, and synaptic plasticity in the CNS (Mursal et al. 2024). Although the peripheral functions of BDNF remain less well characterized, evidence suggests its involvement in nerve regeneration following injury and modulation of immune responses (Mursal et al. 2024). BDNF mainly mediates its effects by binding to the TrkB tyrosine kinase receptor, thereby initiating phosphorylation cascades and activating several intracellular signaling pathways (Sasi et al. 2017). BDNF–TrkB signaling has been shown to modulate NF‐κB expression, thereby contributing to the reduction of neuroinflammation (Hu et al. 2024). Conversely, depletion of BDNF is closely linked to the pathogenic features of AD and PD, including tau hyperphosphorylation, amyloid‐β (Aβ) buildup, neuroinflammation, and neuronal death (Ali et al. 2025). Importantly, stimulation of BDNF–TrkB signaling promotes tau dephosphorylation, offering a potential mechanism for neuroprotection (Gupta et al. 2025).

Clinical studies consistently report altered circulating BDNF levels in patients with AD and PD, although similar alteration have also been described in other neuropsychiatric disorders such as depression, schizophrenia, AD, multiple sclerosis, and anorexia nervosa (Patel et al. 2024; Ng et al. 2019). In AD specifically, both BDNF protein and mRNA levels are downregulated, a phenomenon also observed under chronic stress exposure, whereas antidepressant treatment tends to normalize BDNF expression (Ng et al. 2019). Elevated serum BDNF levels have been associated with a reduced risk of developing AD, highlighting its potential as both a biomarker and therapeutic target (Qin et al. 2017). Importantly, phytochemicals promote the growth of SCFA‐producing gut bacteria, resulting in increased butyrate production (Dong and Cui 2022). Butyrate enhances BDNF signaling through histone deacetylase (HDAC) inhibition and epigenetic upregulation of BDNF expression in hippocampal neurons, thereby promoting neuronal resilience, attenuating neuroinflammation, and supporting neuroprotection in AD and PD via the gut–brain axis (Kumar and Khanum 2012).

#### 3.4.6 Inflammatory Cytokines and Oxidative Stress (OS)

OS and inflammatory cytokines are closely related to the GBA and play a pivotal role in the pathogenesis of AD and PD (Kumar and Khanum 2012). Cytokine‐driven inflammation stimulates ROS production through immune cell activation, particularly via enzymes such as NADPH oxidase and myeloperoxidase, thereby amplifying oxidative damage (Cross et al. 2020; Rahaman et al. 2025b). Conversely, excessive ROS activates NF‐κB, which upregulates proinflammatory cytokines and perpetuates neuroinflammation (Babkina et al. 2021). Pro‐inflammatory cytokines themselves can promote ROS overproduction while simultaneously weakening endogenous antioxidant defenses, further exacerbating oxidative injury (Liu et al. 2023b).). Dysregulation of this cytokine–oxidative stress axis is therefore considered a key mechanism underlying the progression of AD and PD (Reynolds et al. 2007).

Within the context of the GBA, microbial dysbiosis promotes the release of LPS, which subsequently elevates levels of cytokines including IL‐1β, IL‐6, and TNF‐α (Wan et al. 2025a). These mediators compromise intestinal tight junction integrity, facilitating the translocation of inflammatory mediators and ROS‐promoting molecules into systemic circulation (Rahaman et al. 2025b; Wan et al. 2025b). Importantly, phytochemicals remodel the gut microbiota by suppressing LPS producing Gram‐negative bacteria while enriching SCFA‐producing taxa. Consequently, LPS release and pro‐inflammatory cytokine production are reduced, thereby attenuating oxidative stress, restoring redox homeostasis, and limiting neuroinflammation associated with AD and PD via the gut–brain axis (Alarabei et al. 2023).

#### 3.4.7 Autophagy Mechanism of Misfolded Proteins

Aggregation of misfolded proteins is a fundamental pathological feature of AD and PD, including amyloid‐β plaques and tau tangles in AD, as well as α‐synuclein aggregates in PD (Koszła and Sołek 2024). This multistep, dynamic process leads to neuronal dysfunction, which is a hallmark of both AD and PD (Khodosevich and Sellgren 2023).

Autophagy, a critical catabolic process, is responsible for degrading cytoplasmic contents, including misfolded proteins, through the lysosomal system (Kim and Lee 2014). GBA regulates autophagy via metabolites, neurotransmitters, hormones, immunological signals, and neurological pathways (Yao and Han 2025). Proper GBA signaling facilitates effective autophagy in both the gut and brain, whereas dysbiosis, inflammation, or metabolic imbalance can impair autophagy, ultimately contributing to the progression of AD and PD (Jung et al. 2024).

Gut microbiota‐derived modulators of autophagy include SCFAs, neurotransmitters such as serotonin (5‐HT), GABA, as well as proinflammatory cytokines (IL‐1β, TNF‐α, IL‐6) (Muraleedharan and Ray 2024). SCFAs and serotonin inhibit the aggregation of misfolded protein through activation of the AMPK/mTOR and PI3K/Akt signaling pathways (Singh et al. 2024c), respectively, whereas proinflammatory cytokines promote aggregation and impair autophagic flux through NF‐κB activation (Deng et al. 2021; Zhang et al. 2016). Because phytochemicals regulate gut microbiota composition, they indirectly modulate these gut–brain axis mediators, including SCFAs, neurotransmitters, and inflammatory cytokines. Consequently, phytochemical‐mediated remodeling of the gut microbiota promotes autophagy by enhancing beneficial microbial metabolites while suppressing pro‐inflammatory signaling. In addition, phytochemicals directly activate the AMPK/mTOR signaling pathway, facilitating the autophagic clearance of amyloid‐β, tau, and α‐synuclein aggregates, thereby attenuating neurodegeneration in AD and PD (Limanaqi et al. 2019).

#### 3.4.8 Microglia and Astrocytes

Microglia and astrocytes serve as the principle immune defense cells in the CNS, directing neuroinflammatory and homeostatic responses (Carson et al. 2006). Within the GBA, microbial dysbiosis and increased intestinal permeability promote the release endotoxins, including LPS and pro‐inflammatory cytokines into systemic circulation (Li et al. 2021b). These mediators can cross the BBB or impact circumventricular organs, activating microglia, which adopt a pro‐inflammatory phenotype and release cytokines and ROS, leading to neurotoxicity and the aggregation of misfolding protein (Cervellati et al. 2020). Persistent activation of microglial and astrocytic amplifies neuroinflammation, contributing to amyloid‐β and tau pathology in AD and α‐synuclein aggregation in PD (Gao et al. 2023).

Phytochemicals can modulate these glial responses primarily through remodeling of the gut microbiota, thereby reducing microbial dysbiosis, LPS production, and systemic inflammation. Consequently, microglial and astrocytic activation is attenuated, leading to reduced neuroinflammatory signaling within the CNS. Notably, urolithin A, gut microbial metabolite generated from ellagitannins presents in dietary phytochemicals, exerts anti‐inflammatory and neuroprotective effects by activating mitophagy and modulating microglial function (Reynolds et al. 2026). These findings highlight that phytochemical–microbiota interactions regulate glial activation through the gut–brain axis, thereby limiting neuroinflammation and slowing the progression of AD and PD.

### 3.5 Challenges and Limitations

#### 3.5.1 Variability in Individual Gut Microbiomes

The human gut microbiome exhibits substantial variability influenced by multiple host and environmental factors, including dietary patterns, age, sex, genetic background, geographical location, and lifestyle variables such as antibiotic exposure and physical activity (Falony et al. 2016; Lloyd‐Price et al. 2016). This inter‐individual variability represents a major challenge in understanding microbiota‐mediated mechanisms and predicting responses to phytochemical‐based interventions in AD and PD. This heterogeneity manifests both within individuals over time (intra‐individual variability) and across different individuals (inter‐individual variability), reflecting the dynamic nature of microbial ecosystems under changing physiological and environmental conditions (Procházková et al. 2023). Host physiological characteristics, including intestinal transit time and luminal pH, further contribute to microbiome variability by shaping microbial composition and metabolic function (Procházková et al. 2024).

Diet represents one of the most prominent modulators of gut microbial structure. Distinct bacterial taxa, such as *Bacteroides*, *Prevotella*, and *Lactobacillus*, respond differentially to variations in fiber intake and prebiotic consumption, underscoring the diet‐microbiome interaction (Grace‐Farfaglia et al. 2022). Microbial diversity also evolves across the lifespan; *Bifidobacterium* species typically dominate during infancy, whereas members of *Firmicutes* and *Bacteroidetes* become more prevalent in adulthood (Cresci and Bawden 2015). These age‐related shifts are shaped by host genetics and physiological parameters, including gut pH and transit time, which influence taxa such as *Akkermansia* and *Ruminococcus (*Pedroza Matute and Iyavoo 2023).

External perturbations, particularly antibiotic use, can profoundly disrupt gut microbial communities, often leading to reductions in beneficial taxa such as *Bifidobacterium* and *Faecalibacterium (*Cusumano et al. 2025 *)*. Environmental and lifestyle factors, including cohabitation, physical exercise, and alcohol consumption also contribute to alterations in microbial populations, affecting genera such as *Lactobacillus* and *Clostridium* (Cusumano et al. 2025). Disease states further modify gut microbiota composition; for example, inflammatory bowel disease (IBD) is characterized by decreased abundance of anti‐inflammatory species such as *Faecalibacterium prausnitzii* and increased prevalence of opportunistic pathogens including *Escherichia coli* (Lopez‐Siles et al. 2014).

Overall, the highly individualized nature of the gut microbiome presents a significant challenge for developing microbiota‐targeted phytochemical therapies, as differences in microbial composition may influence treatment efficacy and clinical outcomes in AD and PD.

#### 3.5.2 Bioavailability and Metabolism of Plant‐Based Compounds

The biological efficacy of plant‐derived compounds is strongly influenced by their bioavailability and metabolic fate, which are governed by factors such as chemical structure, solubility, and the surrounding food matrix (Manach et al. 2004). These factors represent important challenges for the therapeutic application of phytochemicals in AD and PD, as they determine the concentrations of bioactive compounds that reach both the gut microbiota and the central nervous system. Lipid‐soluble and water‐soluble phytochemicals, including carotenoids and polyphenols, respectively exhibit distinct absorption mechanisms, while the food matrix plays a critical role in modulating their release, stability, and intestinal uptake (Rein et al. 2013). Enzymatic activity, microbial biotransformation, and interactions with dietary constituents further shape the extent of absorption, systemic distribution, and elimination.

The pharmacokinetic behavior of phytochemicals is commonly described by the ADME framework, encompassing release from the food matrix, gastrointestinal absorption, systemic distribution, enzymatic metabolism, and excretion (Velderrain‐Rodríguez et al. 2014). During metabolism, many phytochemicals are converted into more water‐soluble derivatives, such as glucuronide and sulfate conjugates. Importantly, these metabolites may retain or even acquire biological activity, contributing substantially to the overall physiological effects of the parent compounds (Cardona et al. 2013). Gut microbiota also plays a pivotal role in this process by biotransforming phytochemicals into metabolites with enhanced bioavailability or biological activity, thereby influencing their therapeutic efficacy through the gut–brain axis.

Given the intrinsic limitations associated with phytochemical bioavailability, various technological strategies have been developed to enhance their delivery and efficacy. Approaches such as nanotechnology‐based formulations, colloidal dispersions, and food processing techniques, including fermentation have demonstrated considerable potential in improving compound stability and intestinal absorption (Alam et al. 2023; Ramakrishna et al. 2021). Additionally, co‐administration with lipids or specific dietary components can further optimize bio accessibility and maximize health benefits (Mashurabad et al. 2017). Improving phytochemical bioavailability and microbiota‐mediated metabolism is therefore expected to enhance therapeutic outcomes in AD and PD by strengthening gut–brain axis signaling and increasing the delivery of neuroprotective metabolites.

#### 3.5.3 Challenges in Translating Preclinical Findings to Humans

One of the major challenges in developing phytochemical‐based therapies for AD and PD. Although animal models, have provided valuable mechanistic insights into gut microbiota–brain interactions and the neuroprotective effects of phytochemicals, they often fail to fully recapitulate the complexity of human disease and physiology (Marshall et al. 2023). Human AD and PD are inherently complex, characterized by substantial heterogeneity across patients, tumors, and microenvironments, which complicates the extrapolation of preclinical outcomes. These discrepancies may lead to inaccurate estimations of therapeutic efficacy, resulting in either underestimation or overestimation of treatment effect (Lowenstein and Castro 2009). Furthermore, deficiencies in experimental design significantly compromise the reliability of preclinical evidence.

Systematic bias represents another critical limitation. Inadequate reporting practices, insufficient controls, and failure to account for confounding variables, including diet, physical activity, gut microbial composition and housing conditions reduce reproducibility and weaken the interpretability of findings (Seyhan 2019). These challenges are often compounded by suboptimal data processing and inappropriate statistical methodologies, further undermining the robustness of experimental conclusions (Dhir et al. 2020). Consequently, inconsistencies across preclinical studies have contributed to declining confidence in translational research (Marshall et al. 2023).

Environmental variability also exerts a significant influence on experimental outcomes. Even subtle differences in laboratory conditions, such as growth media composition, animal husbandry or cell culture techniques, can introduce variability and hinder comparisons with human biology (Chamuleau et al. 2018). In parallel, intrinsic biological differences across animal models impose fundamental constraints on predictive accuracy (Seyhan 2019). Moreover, substantial differences in gut microbiota composition between laboratory animals and humans may alter phytochemical metabolism, bioavailability, and therapeutic responses, thereby limiting the clinical translation of microbiota‐targeted interventions.

Finally, preclinical research is constrained by both practical and ethical considerations, which limit the extent to which experimental conditions can be replicated in human studies (Lowenstein and Castro 2009). These challenges contribute to the well‐recognized “Valley of Death” in translational science, wherein numerous promising preclinical discoveries fail to progress into clinical success, resulting in substantial attrition throughout the drug development pipeline (Seyhan 2019). Future studies should integrate standardized experimental protocols, humanized microbiome models, and well‐designed clinical trials to improve the translation of phytochemical‐based gut–brain axis interventions for AD and PD.

### 3.6 Future Directions

Future research should emphasize rigorous mechanistic validation to delineate causal relationships between phytochemical‐induced gut microbiota modulation and neuroprotective outcomes in AD and PD. The application of germ‐free models, fecal microbiota transplantation, humanized microbiome models, and integrated multi‐omics frameworks will be particularly valuable for disentangling host‐microbiome interactions and identifying functional pathways underlying therapeutic effects. Concurrently, greater standardization of microbiome sequencing methodologies and experimental protocols is required to improve reproducibility and cross‐study comparability.

A deeper characterization of microbiota‐derived metabolites, including SCFAs and tryptophan metabolites, remains essential for understanding the molecular mediators linking gut ecology with neural function. Future studies should also investigate how phytochemicals are biotransformed by the gut microbiota into bioactive metabolites and how these metabolites influence gut–brain axis signaling, neuroinflammation, autophagy, and protein aggregation in AD and PD. In parallel, comprehensive evaluation of the pharmacokinetics, bioavailability, and microbiota‐dependent biotransformation of phytochemicals is necessary to strengthen translational relevance and optimize therapeutic strategies.

Importantly, well‐designed clinical investigations incorporating longitudinal microbiome profiling, inflammatory biomarkers, and neurobehavioral endpoints are needed to determine whether microbiota‐targeting natural compounds can yield consistent and durable benefits in patients with AD and PD. Addressing key translational variables such as dose optimization, identification of responder‐specific microbiome signatures, and potential synergistic integration with existing pharmacotherapies will be critical for advancing microbiota‐based interventions. Ultimately, integrating phytochemical therapy with precision microbiome‐based approaches may provide personalized therapeutic strategies for modulating the gut–brain axis and slowing disease progression in AD and PD.

## 4 Conclusion

This review consolidates growing evidence that phytochemicals‐mediated modulation of the gut microbiota constitutes a unifying mechanistic framework linking dietary phytochemicals to neuroprotection in AD and PD. Despite substantial structural heterogeneity among the phytochemicals discussed, including flavonoids, alkaloids, polysaccharides, and fermented products, their biological effects consistently converge on core GBA pathways. Across experimental models of AD and PD, restoration of microbial homeostasis was repeatedly associated with reduced endotoxemia, suppression of TLR4/NF‐κB‐mediated neuroinflammation, attenuation of NLRP3 inflammasome activation, reinforcement of intestinal barrier integrity, and activation of neurotrophic signaling networks, including BDNF‐dependent pathways. Phytochemicals also promote the growth of beneficial gut bacteria and increase the production of SCFAs, which regulate immune responses, maintain intestinal barrier function, and modulate gut–brain axis signaling, thereby contributing to neuroprotection. Overall, the findings highlight the bidirectional crosstalk between phytochemicals and the gut microbiota as a promising therapeutic strategy for modulating the gut–brain axis and slowing the progression of AD and PD. Nevertheless, additional mechanistic studies and well‐designed clinical trials are required to validate these findings and facilitate the translation of microbiota‐targeted phytochemical interventions into clinical practice.

## Author Contributions

**Md Emon:** conceptualization, methodology, formal analysis, investigation, data curation, visualization, writing – original draft. **Md Mizanur Rahaman:** conceptualization, investigation, writing – original draft, visualization, writing – review and editing. **Dhrubo Sarder:** investigation, data curation, methodology, writing – review and editing. **Md Shadin:** validation, visualization, writing – review and editing. **Shamima Akter Neela:** validation, data curation, writing – review and editing. **Phurpa Wangchuk:** supervision, writing – review and editing. **Subir Sarker:** conceptualization, writing – review and editing, project administration, supervision, resources, funding acquisition, software.

## Declaration of Generative AI and AI‐Assisted Technologies in the Writing Process

During the preparation of this work the authors used ChatGPT to improve language and readability. After using this tool/service, the authors reviewed and edited the content as needed and take full responsibility for the content of the publication.

## Funding

The authors have nothing to report.

## Ethics Statement

The authors have nothing to report.

## Conflicts of Interest

The authors declare no conflicts of interest.

## Data Availability

No data was used for the research described in the article. Data sharing not applicable to this article as no datasets were generated or analyzed during the current study.
